# The use of discrete choice experiments to inform health workforce policy: a systematic review

**DOI:** 10.1186/1472-6963-14-367

**Published:** 2014-09-01

**Authors:** Kate L Mandeville, Mylene Lagarde, Kara Hanson

**Affiliations:** Department of Global Health and Development, London School of Hygiene and Tropical Medicine, 15-17 Tavistock Place, London, WC1H 9SH UK

**Keywords:** Discrete choice experiment, Stated preferences, Human resources for health, Health workers, Health professionals

## Abstract

**Background:**

Discrete choice experiments have become a popular study design to study the labour market preferences of health workers. Discrete choice experiments in health, however, have been criticised for lagging behind best practice and there are specific methodological considerations for those focused on job choices. We performed a systematic review of the application of discrete choice experiments to inform health workforce policy.

**Methods:**

We searched for discrete choice experiments that examined the labour market preferences of health workers, including doctors, nurses, allied health professionals, mid-level and community health workers. We searched Medline, Embase, Global Health, other databases and grey literature repositories with no limits on date or language and contacted 44 experts. Features of choice task and experimental design, conduct and analysis of included studies were assessed against best practice. An assessment of validity was undertaken for all studies, with a comparison of results from those with low risk of bias and a similar objective and context.

**Results:**

Twenty-seven studies were included, with over half set in low- and middle-income countries. There were more studies published in the last four years than the previous ten years. Doctors or medical students were the most studied cadre. Studies frequently pooled results from heterogeneous subgroups or extrapolated these results to the general population. Only one third of studies included an opt-out option, despite all health workers having the option to exit the labour market. Just five studies combined results with cost data to assess the cost effectiveness of various policy options. Comparison of results from similar studies broadly showed the importance of bonus payments and postgraduate training opportunities and the unpopularity of time commitments for the uptake of rural posts.

**Conclusions:**

This is the first systematic review of discrete choice experiments in human resources for health. We identified specific issues relating to this application of which practitioners should be aware to ensure robust results. In particular, there is a need for more defined target populations and increased synthesis with cost data. Research on a wider range of health workers and the generalisability of results would be welcome to better inform policy.

**Electronic supplementary material:**

The online version of this article (doi:10.1186/1472-6963-14-367) contains supplementary material, which is available to authorized users.

## Background

The global inequities in health worker numbers and distribution have been well-described [1–3]. Yet there has been less focus on the tools available to inform the policy mechanisms to improve this situation [4]. Information systems for tracking health workers are weak in many countries, impeding longitudinal studies [1, 2]. Qualitative surveys can identify preferred job characteristics but not the relative strength of these preferences [5, 6]. Political, ethical and logistical factors limit the opportunities for natural or controlled experiments [4, 7]. In light of this limited toolkit, one approach has become increasingly popular amongst researchers in this area: the discrete choice experiment (DCE).

DCEs are a quantitative technique for eliciting preferences [8–10]. They are based on Lancaster’s theory that goods and services can be described by their essential characteristics and the value of a good or service to an individual is derived from the combination of these characteristics [11]. In a DCE, participants are presented with descriptions of hypothetical goods and services based on a combination of characteristics and asked to select their preferred option. Thus DCEs provide “stated” preference data as opposed to the “revealed” preference data derived from empirical studies examining actual choices [12]. The use of DCEs was pioneered in the fields of market research, transport and environmental economics before being used to explore preferences in health services [13–21]. More recently, they have been applied to the study of labour market decisions and preferences of health workers [22].

**Figure 1:**
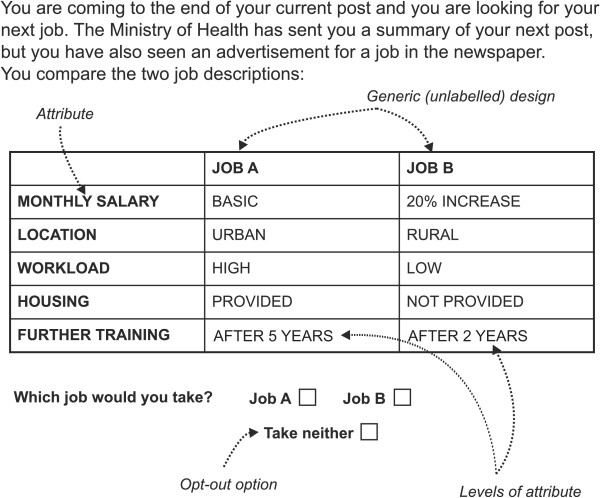
**An example choice task in a discrete choice experiment.**

In DCEs in health workforce research, participants are usually asked to select between different choice profiles that read like hypothetical job descriptions. Each profile is made up of several attributes which describe the job in question (for example, “salary” or “location”) and each attribute takes one of several possible levels (e.g. “salary” could take the levels “basic”, “20% increase” or “50% increase”). Choice profiles are usually combined to form choice tasks, in which participants are asked to select their preferred profile (Figure 1 presents an example and key terms used in this review). Participants’ choices over a number of alternatives can be analysed to deduce the relative importance of these attributes [22]. DCEs have two main advantages as a methodology over revealed preference data. Firstly, a wide range of attributes can be included in the job descriptions, including some not yet offered. Thus, health worker preferences can be elicited beyond the current situation, and jobs that respond more fully to these preferences can be modelled [23]. Secondly, revealed preference data often display multicollinearity between independent variables, where the most popular jobs are the ones with the best salaries, the best working conditions, and the best locations [24]. In a DCE, the researcher constructs the job descriptions based on an experimental design so that the effect of each individual attribute can be independently assessed in statistical analysis.

A narrative literature review conducted by Lagarde and Blaauw in 2008 found ten studies that employed DCEs to examine health workers’ preferences [22]. Since then, two global forums on human resources for health (HRH) have advocated for more research to inform policy on health workers [25, 26], a “Rapid DCE” tool has been developed for use in low-income countries [27] and a user guide for conducting DCEs in HRHs for non-specialist practitioners has been published [28]. Yet the dissemination of DCEs as an accessible tool in HRH research may have been at the expense of maintaining methodological robustness. DCEs as a technique are evolving rapidly, with ongoing methodological debates and research [29–31]. DCEs in health economics have been criticised in the past for lagging behind current best practice in other fields of economics, limiting the validity of their results [31–33]. The Lagarde-Blaauw review found that all studies but one used non-optimal experimental designs [22]. In contrast, a 2012 review by de Bekker-Grob *et al.* [30] compared DCEs in health economics published between 2001–2008 to a previous review conducted by the same group between 1990 and 2000 [34]. They found a shift towards more statistically efficient designs and less restrictive econometric models. However, this review only included five of the DCEs identified by Lagarde and Blauuw, with no detailed analysis of health workforce issues. Due to the rapid developments in this application of DCEs and with renewed focus on health worker shortages due to the universal health coverage agenda, we considered it timely to systematically review the use of DCEs in health workforce policy.

## Methods

### Search terms

The scope of the review was discrete choice experiments looking at the job preferences of health workers, including doctors, nurses, allied health professionals such as pharmacists, mid-level cadres such as clinical officers, and community health workers. All low-, middle- and high-income countries were included, and there were no limits on date or language.

Search terms were: “health*worker* OR health* personnel OR health* professional* OR human resource* OR staff OR doctor* OR physician* OR clinical OR medic* OR nurse OR midwi?e* OR pharmacist*” AND “discrete-choice* OR choice experiment* OR stated preference* OR job preference* OR conjoint analysis”.

### Search strategy

We searched the following six databases in order to achieve comprehensive coverage of the healthcare, global health and economics literature: Medline, Embase, Popline, Global Health, Econlit, and Social Policy & Practice. We also searched three grey literature repositories: the HRH Global Resource Center (http://www.hrhresourcecenter.org/), the Global Workforce Alliance Knowledge Centre (http://www.who.int/workforcealliance/knowledge/en/), and the National Bureau of Economic Research Working Papers (http://www.nber.org/papers.html). A search was also undertaken for us of a database of studies collated by the University of Southampton (United Kingdom) on the use of DCEs in health.

The titles and abstracts of identified studies were screened for relevance. The full text of relevant studies was assessed for eligibility. Ambiguous cases for inclusion were discussed between two of the authors. References of included studies were checked for further relevant studies.

### Contact of experts

In order to identify studies not yet included in databases, we contacted experts in the field. These included the corresponding authors of all studies identified by the earlier review and a number of other researchers known to be involved in DCE work. Forty-four experts were contacted, with one reminder email sent after four weeks.

### Assessment of included studies

#### Review of study characteristics

We followed a framework consisting of the four main stages of a DCE (choice task design, experimental design, conduct and analysis) to construct and pilot forms to extract data for key characteristics of included studies. We took the date of publication as that of the earliest publication of the study, in order to more closely reflect when studies were carried out rather than the delays in the publication process. In contrast, if information differed between versions, we used data contained in the peer-reviewed publication where available.

#### Assessment of validity

We collated a list of 13 criteria to assess the validity of included studies, here defined as the risk of bias or systematic error (see Additional file 1). We drew on a comprehensive quality checklist constructed by Lancsar and Louviere [29], as well as areas of concern highlighted by previous reviews [30]. As quality checklists are poorly correlated with validity of studies and often measure the quality of reporting rather than that of the underlying research [35, 36], we limited these criteria to those we considered a substantive threat to the validity of results. These covered all four key stages of a DCE, as poor validity in one stage cannot be negated by high validity in another. Justification for the choice of these criteria is included in Additional file 1. We assessed whether each criterion for each study was met or not. If the information available for a criterion in any of the study publications was insufficient to judge its achievement, we noted this as a separate category.

#### Comparison of results

With the increasing number of health workforce DCEs, it would be useful to compare results from studies with similar aims in order to draw broad conclusions from the growing evidence base. Unfortunately, generalisation beyond a single DCE is challenging. It is not possible to directly combine the results of econometric estimations from different studies as coefficients of attributes within a study are interdependent, so to display coefficients from different studies on a linear scale would be misleading [22]. In addition, differences in coefficients from separate datasets may be due to scale variance rather than true differences [4]. It is more appropriate to compare the relative impact of different attributes across studies when the coefficients have been transformed by methods such as marginal willingness-to-pay or probability analyses.

Only studies that met more than three quarters of the validity criteria (10 out of 13) were included in this comparison. This threshold is necessarily arbitrary when the validity of studies is better thought of as a spectrum [35], however this restricted the comparison of results to those studies with few threats to the validity of their results. We compared willingness-to-pay estimates or probability analyses from studies with homogeneous objectives and similar contexts.

No ethical approval was required for this study.

## Results

### Included studies

**Figure 2:**
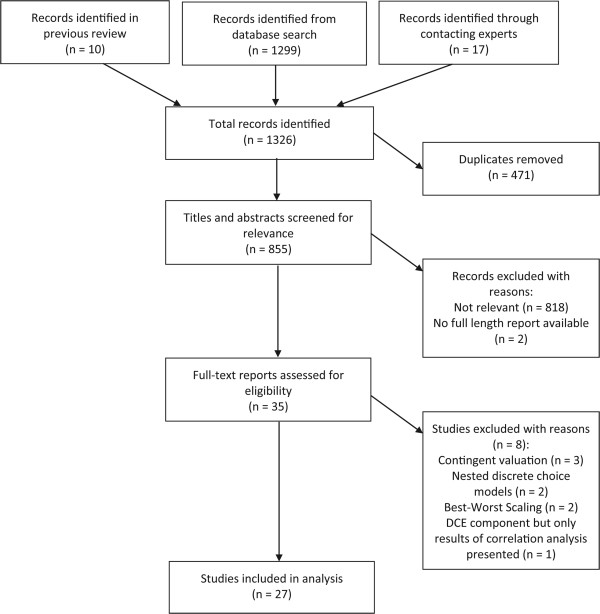
**Flow of studies.**

Figure 2 details the flow of papers through the study. In total, 1326 records were identified through searching databases and contacting experts. Thirty-one out of 44 experts replied to our survey, a response rate of 70.5%, identifying 17 additional studies. From those screened as relevant, two studies were excluded as no full length report was available despite contacting the authors. Eight studies were excluded as their design or analysis were not discrete choice experiments [37–44]. In total, 27 studies were included: ten identified by the previous Lagarde-Blaauw review and 17 new studies.

### Review of included studies

**Figure 3:**
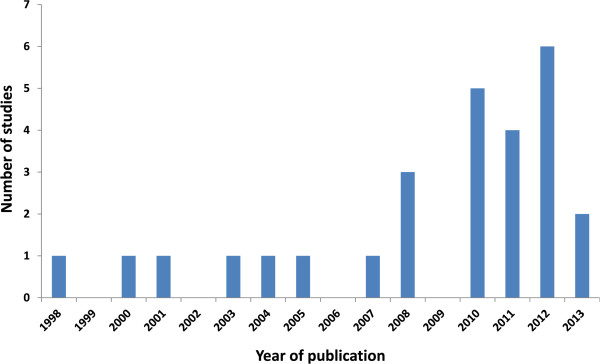
**Publication date of included studies.**

Here we review key study characteristics, commenting on specific methodological debates for this application of DCEs (details of studies and key characteristics are included in Additional file 2). Overall, there were more DCE studies published in the last four years than between 1998 and 2009 (Figure 3). In 2012 alone, there were six new studies.

The majority of new studies (15/17) have been carried out in low and middle income countries (LMIC). In contrast, the Lagarde-Blaauw review found the number of studies carried out in high income countries (HIC) equalled those carried out in LMIC [22]. With over 80% of all DCEs set in LMIC (15/18) published since 2010, the call to produce more evidence for health workforce policy is clearly being heeded. The most common objective was to explore health worker preferences for working in rural and/or remote areas, examined in 17 studies with 16 of these set in LMIC.

Doctors and medical students were the focus of two thirds of DCE studies (66.7%, 18/27) [5, 23, 45–60]. Two studies [51, 58] were from a large longitudinal study of the employment preferences of Australian doctors known as MABEL (“Medicine in Australia: Balancing Employment and Life”). In contrast, mid-level cadres such as clinical officers [6] and medical assistants [59] were the focus of one study each, even though these cadres may present a more cost-effective response to health worker shortages, particularly in rural or remote areas. Moreover, no study has yet focused on community health workers, who as mostly volunteer workers may have very different preferences to salaried health professionals.

Students training to be health workers were included as participants in nearly half of all studies (44.4%, 12/27). No study set in a HIC contained just students as participants, compared to seven in LMIC. Undoubtedly, students offer more convenient survey administration, with relatively large populations in a limited number of locations that are far easier to convene than practicing health workers. Yet with most studies aiming to inform policy for practicing health workers, the extrapolation of utility values from students is concerning. Students nearing the end of their course were often targeted with the justification that they would soon graduate and select jobs based on their current preferences. Even students nearing the end of their training, however, are likely to hold different preferences to qualified workers who have managed a job and salary under prevailing working conditions. For example, Vujicic *et al.* [61] found that the location of workplace (rural/urban) was the most important attribute for doctors in a DCE undertaken in Vietnam, whereas it was long-term education for medical students. Moreover, there were five fold differences between doctors and medical students in willingness-to-pay estimates for some job attributes. Rockers *et al.* found similar differences in preferences for attributes of rural jobs between practising nurses and nursing students in Laos [62]. And whilst the target population is often students nearing graduation, shortfalls in recruitment can lead to students from earlier years being included, increasing the disparity in experiences [59]. Finally, two studies pooled results for students and graduates from the same cadre for at least part of the analysis [53, 59]. This is likely to lead to less valid results and overestimation of the willingness of qualified health workers to accept certain conditions.

#### Choice task design

A third of studies (33.3%, 9/27) identified attributes and levels through a combination of literature/policy reviews and qualitative work with target participants and policymakers, which is best practice to obtain valid and policy-relevant attributes [63, 64] (Table 1). The vast majority (85.2%, 23/27), however, conducted some qualitative work (focus groups or interviews) with representatives of the target population. This is important to ensure the attributes and levels chosen are salient to the target population, encouraging engagement with the choice task presented [29].

**Table 1:** **Choice task design of included studies**

Design aspect	Specification	Number of studies (%)
**Preparatory work**	Literature review	20 (74.1)
	Participant qualitative work	23 (85.2)
	Policymaker qualitative work	16 (59.3)
	All three methods	10 (37.0)
**Type of choice**	Binary	21 (77.8)
	Ternary	1 (3.7)
	Quaternary	2 (7.4)
	Mixed binary/ternary	3 (11.1)
**Attributes**	5	3 (18.5)
	6	8 (29.6)
	7	12 (44.4)
	8	4 (14.8)
**Labelling**	Generic	20 (74.1)
	Labelled	7 (25.9)
**Opt -out option**	Yes	8 (29.6)
	No	19 (70.4)

Three out of four studies (77.8%, 21/27) presented a binary choice task to participants, with only three studies using higher-order choices of ternary [53] and quaternary [57, 65] choices. Yet labour markets for health workers are complex [66]. Along with the option to remain in their current job, health workers can internally migrate between locations or sectors or overseas, the latter of particular concern in LMIC. In a novel approach, Lagarde *et al.* [65] presented four labelled profiles in different sectors and locations to South African nurses: overseas, public rural, public urban, and private urban. Although there is evidence that increasing task complexity (such as adding more alternatives) can decrease quality of choice responses [29, 67], the cognitive dissonance created by a less realistic representation of the job market available to participants may in itself produce less valid choices.

Choice tasks can also include an *opt-out*, in the form of a “choose none” or a *status quo* (“choose my current job”) option [29]. Nearly one in three studies in this review (8/27, 29.6%) included such an option, compared to just one in the Lagarde-Blaauw review. Three studies presented a two stage choice to participants, one as a forced binary choice between two presented profiles and one ternary choice containing an opt-out [68–70]. The inclusion of an opt-out option can avoid a “forced choice” which assumes that one of the alternatives offered must be taken up and may falsely increase the strength of preference associated with alternatives, distorting related welfare estimates [29, 31, 71–74]. Indeed, the instruction to “assume these are the only options available to you” is a common way of framing a choice task. In real life, however, health workers always have many options in the labour market, including the *status quo* of staying in their current job or withdrawing from the health labour market altogether. This holds true even for students or new graduates. Although consumption of the good or service on offer can rarely be assumed in DCE applications in health, except for perhaps comparing new treatments versus current treatments, it is arguably more pertinent here. After all, labour market decisions are complex decisions with significant consequences, frequently associated with major disruptive effects on an individual’s status quo, and the total number made over a lifetime is comparatively few compared to other types of decisions. Maintaining this status quo by opting out of a choice between job profiles may seem very attractive, and its inclusion more closely reflects the real world market. This is especially important for measures of relative attribute impact such as willingness to pay for desirable job characteristics (see below). The disadvantage is that the researcher risks not obtaining sufficient information on preferences to estimate the analytical model if an opt-out option is chosen by the majority of participants. The use of a two stage choice, with both a forced choice and a choice with an opt-out option, seems pragmatic until sufficient information is gleaned on the likely distribution of responses. Scott *et al.* used this approach for a DCE on Australian GPs embedded within the MABEL survey [70], but went on to construct the status quo for each participant through responses to other questions gathered in the larger survey. This innovative use of accompanying survey data meant that no information was lost when participants chose the status quo option, as attributes and levels for this alternative could be defined on an individual level. If the status quo varies within the target population, then participants should be asked to identify their status quo through survey questions in order to model these alternatives [29]. Researchers should be careful to frame the choice task in a way that does not downplay the opt-out option, in order to increase accuracy of welfare estimates.

Choice tasks profiles can be *generic*, e.g. “Job A” versus “Job B”, or *labelled* e.g. “Rural clinic” versus “Urban hospital” (Figure 1). Generic designs were used by the majority of studies (74.1%, 20/27), although seven studies featuring a labelled design in the last three years [4, 52–54, 57, 65, 69]. All of these studies presented rural versus urban alternatives, except the above study by Lagarde *et al.* that also included jobs overseas and in private facilities [65]. The use of labelled designs in this way can enhance realism for participants by allowing alternative-specific attributes to be defined in order to avoid unrealistic combinations that might lead to participant confusion and/or disengagement with the questionnaire (for example, the availability of private practice in rural posts) [4, 54, 56, 75]. Labelled designs can also provide choices between additional qualities associated with the labels by participants, but not captured by the limited number of attributes [75]. The drawback is that these qualities are not delineated, so researchers cannot be certain if their interpretation of the label matches that of the participants. In addition, label-specific attributes/levels are correlated with the label, and therefore their utilities cannot be distinguished in the analysis [75]. This may not be a disadvantage, however, if the policy aim is to investigate preferences for specific job types in a given market (e.g. rural/urban/overseas) or how individuals value the same attribute in different posts. In contrast, a generic choice is more appropriate where the research interest is the trade-off between different attributes for one particular type of job.

#### Experimental design

The assessment of experimental design was hampered by poor reporting (Table 2). All studies used a fractional factorial design to decrease the total number of possible attribute and level combinations to a more manageable number, with SAS software (http://www.sas.com, 40.7%, 11/27) the most popular design source. Only one study reported using interaction terms within its fractional factorial design so as to be able to identify the modification of the preference for one attribute based on the level of another [6], with the vast majority (88.9%, 24/27) assessed as including main effects only (the primary effect of each attribute). The inclusion of interaction terms increases the number of choice tasks required to make accurate estimates [28, 29] and it is not common practice in health economics DCEs, with only 5% of studies including two-way interactions between attributes in the Bekker-Grob review [30]. Yet preferences for attributes of health workers’ jobs may well depend on the level of other attributes. For example, free transport may be more highly valued in a rural area than an urban post. Thus it is likely to be inaccurate, albeit pragmatic, to assume that the main effects of attributes are not confounded by each other. The inclusion of selected interaction terms in design plans should be encouraged, based on those that are most likely to be conceptually valid.

**Table 2:** **Experimental design of included studies**

Design aspect	Specification	Number of studies (%)
**Design plan**	Main effects only	4 (14.8)
	Main effects + interactions	1 (3.7)
	Not clearly reported in text but main effects only in primary analysis	20 (74.1)
	Not reported and unclear from analysis	2 (7.4)
**Design source**	SAS	11 (40.7)
	Sawtooth Software	5 (18.5)
	SPEED	3 (11.1)
	IBM SPSS Statistics	2 (7.4)
	Sloane’s orthogonal array	1 (3.7)
	Not reported	5 (18.5)
**Design of choice tasks**	Orthogonal array (all using one constant comparator)	8 (29.6)
	Efficient design	15 (55.6)
	Not clearly reported	4 (14.8)
**Number of choice tasks**	<10	8 (29.6)
	10-15	6 (22.2)
	16-20	13 (48.1)

SPEED = Stated Preference Experiment Editor and Designer.

The majority of studies (55.6%, 15/27) used an efficient design to design their choice tasks, including every study from 2010 onwards that reported design type bar one [60]. This uses an algorithm to maximise the statistical efficiency of the design, and corroborates the increase in this design approach identified by de Bekker-Grob *et al.* Eight studies (29.6%) employed an orthogonal design, which uses an orthogonal array to generate choice profiles and then one of several methods to allocate profiles to choice tasks [10]. In all these studies, a constant comparator approach was used to construct choice tasks, whereby one profile is selected to be paired in each choice task against the remaining choice profiles. This is in contrast to de Bekker-Grob *et al.*, who found just one in three studies using orthogonal arrays using this approach. Its popularity here may be an attempt by researchers to represent a *de facto* status quo option, with one choice profile used to correspond to the prevailing or baseline job conditions. This approach, however, is inefficient and discards much information on choices between attributes, rather than using a constant “neutral” opt-out alternative [22].

Efficient designs also have the advantage of being able to incorporate prior estimates of parameter values rather than setting these at zero. This increases the efficiency of the design through a Bayesian approach, with estimates usually obtained through pilot studies [30, 51]. In contrast to de Bekker-Grob *et al.* who found no studies employing this feature, two health workforce DCEs incorporated priors from a pilot survey, both from the MABEL survey [51, 58]. Given that the limited number of health workers in LMIC and the logistical difficulty of administering surveys to practising health workers, practitioners should consider the use of priors to order to increase the precision of value estimates for small sample sizes [30].

Nearly half the studies (48.1%, 13/27) presented between 16 and 20 choice tasks to participants, with a mean of 12. Blocking was employed by ten studies, usually to decrease the number of choice tasks to less than ten. The number of choice tasks presented to participants is usually restricted due to fears over choice complexity and cognitive burden that may reduce the quality of responses [29]. Amongst a target population that has uniformly completed tertiary education courses characterised by frequent testing, however, higher numbers of choice tasks may be handled without any ensuing loss of engagement. It would be interesting to compare the responses from the same group of health workers to varying number of choice tasks.

#### Conduct

Three quarters of studies (20/27, 74.1%) reported piloting their surveys before full rollout. There was great variation in piloting, however, with pilots ranging from a small focus group of one subgroup within the target population [59] to a four stage procedure with a final random sample of 1091 participants [70]. Piloting is an important part of DCEs, allowing verification of presentation, comprehension, coverage of attributes and levels, complexity, likelihood of the selection of an opt-out option, and data collection for priors as discussed above [29]. The development of a standard checklist for piloting DCEs would be worthwhile, allowing for contextual differences. In particular, pilots should attempt to include representatives from all subgroups of health workers to be analysed in the final sample (e.g. differences in gender, locations, seniority) to ensure that differences in understanding are not leading to variation in preferences associated with these subgroups.

The mode of administration of DCEs is likely to be important both for the response rate and understanding of the task (Additional file 2). Seven studies used postal surveys to contact large numbers of health workers, all in HIC [5, 23, 47, 48, 51, 70, 76]. Two of these studies also included online questionnaires [51, 70], although three studies used computer-assisted surveys on student populations in LMIC [45, 56, 77]. In LMIC, response rates were generally very high, with a mean of 83.2% (range 65.2% to 100%, the latter from a study set in China as reported by authors [60]), compared to 49.3% (16.8 – 65.0%) in HICs. Unsurprisingly, response rates were significantly lower for graduates (mean of 62.7%, range 16.8 – 100%) than for students (mean 84.1%, range 62.7 – 100%), underscoring the potential for distortion if results from these two subgroups are combined. Surveys were most commonly self-administered with supervision by researchers (10/27, 37.0%), a format that allows participants to ask questions for clarification but complete the survey in their own time.

Total sample sizes (Additional file 2) ranged from 102 doctors in Peru [57] to 3727 general practitioners in Australia [58]. Whilst sampling follows the same principles as for other primary data collection i.e. ensuring the sampling frame and sampling strategy are representative of the target population(s), sample size calculation is an ill-defined area within discrete choice experiments. Although various rules of thumb were formed from modelling experience [8, 29], these have become less relevant with the advent of efficient designs that can take into account limited sample sizes [63]. Indeed, a very large sample encompassing wide variability in preferences may lead to less precise results than a small, more homogeneous sample [63]. For health workers, more attention should be placed on the representativeness of the sampling frame in order to extrapolate results to the general population, and the sampling strategy to ensure adequate size of subgroups if significant *post hoc* analysis by different characteristics is planned [29, 63].

#### Analysis

For a succinct summary of modelling approaches to health DCEs, see de Bekker-Grob *et al.* [30] and Amaya-Amaya *et al.* [63]. While most studies pre-2010 relied on random effects probit or logit models [63], mixed logit has been the most common econometric model more recently, used in 11 studies (39.3%) after 2010 (Table 3). Mixed logit relaxes the restrictive assumptions of the commonly used multinominal logit model by allowing for heterogeneity of preferences for attributes between participants, which is likely to be high in the fairly diverse health worker populations covered by many of these studies. It does this by introducing an individual-level utility estimate for each attribute calculated from the mean utility estimate for that attribute and an individual-specific deviation from the mean [29, 70]. Although flexible, the mixed logit model has a number of challenges, such as the choice of parameters to define as random. Moreover, the size of these individual-specific variances are likely to vary within and between participants, reducing the precision of utility estimates rather than increasing it. The latent class model has the same advantage over the multinominal logit as mixed logit, however assumes that there are two or more classes (or groups) of participants underlying the data with more homogeneous tastes. The distribution of participants belonging to these classes is not known to the researcher, but is assumed to be related to observed variables such as attitudes and/or socio-demographic characteristics [63]. Latent class models have been used only rarely in health DCEs, with none from this review and just one in de Bekker-Grob *et al.* [30], however this model offers much to health workforce DCEs. As described earlier, quite heterogenous populations are typically included in health DCEs, for which latent class models may be able to separate into subgroups with more similar (and accurate) preferences depending on characteristics, for example years of work experience or growing up in a rural area. Four studies (14.8%) used an extension of mixed logit, generalised multinomial logit models, with three of these finding a better fit to data than comparator mixed logit or logit models [51, 54, 58, 62]. Generalised multinomial logit models are able to account for scale heterogeneity of preferences as well as taste heterogeneity, i.e. utility estimates might vary between individuals not only because of differences in preferences, but also due to differences in variance. Some individuals may be much more certain of their choice than others or use decision heuristics that reduce variance, whilst other participants may not understand the task well or make mistakes that increase variance [70]. Fiebig *et al.* [78] assert that this model can better account for responses from these “extreme” participants, providing an improved fit to the data. This is undoubtedly an attractive feature for DCEs examining labour market decisions (where participants may be more uncertain) in populations of workers that are typically time-poor and highly pressurised (thus perhaps more likely to employ decision heuristics or make mistakes). This may explain its popularity here, with four studies employing it compared to none in de Bekker-Grob *et al.* [30].

**Table 3:** **Analysis of included studies**

Analytic aspect	Specification	Number of studies (%)*
**Econometric model**	Probit	1 (3.7)
	Logit	2 (7.4)
	Random effects probit	7 (25.9)
	Multinomial logit	1 (3.7)
	Conditional logit	3 (11.1)
	Mixed logit	11 (40.7)
	Generalised multinomial logit	4 (14.8)
	Errors component mixed logit	1 (3.7)
**Analysis software**	Stata	16 (59.3)
	NLogit/LIMDEP	5 (18.5)
	SPSS	2 (7.4)
	Not reported	4 (14.8)
**Relative attribute impact analysis**	Probability analysis	16 (59.3)
	Welfare measures	12 (44.4)
	Marginal rates of substitution	5 (18.5)
	Partial log-likelihood analysis	1 (3.7)
	Compensating differentials	1 (3.7)
	Wage equivalents	1 (3.7)
	None	2 (7.4)

*Total for each category greater than total number of studies as some studies used more than one econometric model or relative attribute impact analysis.

As the importance of different attributes cannot be compared directly using parameter estimates due to confounding with the underlying utility scales, the relative impact of attributes is usually examined by converting estimates to a common scale [79]. There are a number of methods to do so, including probability analysis, welfare measures and marginal rates of substitution. Probability analysis and welfare measures were the most popular methods in this review, with 16 (59.3%) and 12 (44.4%) studies employing them respectively. It is surprising that more studies did not calculate welfare measures, given all studies included a monetary variable. Ten out of these 12 studies (83.3%) did not include an opt-out/status quo option, however, which as discussed above is likely to distort welfare measures due to the overestimation of preferences resulting from a forced choice [29]. Despite over half of studies including a time variable, no study presented a marginal rate of substitution for time, in the form of willingness to commit to a post for a defined period. This is an important metric for policymakers, with pragmatic retention policies and incentive packages designed in the knowledge that filling unattractive posts may be for a limited period only.

Nearly all studies using welfare measure(s) framed these as willingness to pay, either marginal (for changes in attributes) or total (for certain alternatives or scenarios). Willingness to pay for health workforce DCEs is rooted in the labour economic theory of compensating wage differentials, which puts forward that differences in wages arise to compensate workers for nonwage characteristics of jobs, for example risk or lack of social amenities [47, 80]. In health workforce DCEs, negative willingness to pay represents the additional amount of income required to compensate a health worker for a job with negative characteristics. For example, Scott *et al.* [70] modelled a range of unattractive job postings with accompanying negative total willingness to pay values. Conversely, positive willingness to pay is the amount of income that a health worker would forego in order to take up a job with desirable characteristics. For example, Vujicic *et al.* [50] estimated the marginal willingness to pay by doctors in Vietnam for various desirable job characteristics, such as urban location and adequate equipment.

However, two thirds of these studies (66.7%, 8/12) used a current income level accompanied by either actual or percentage increases on this baseline. The negative willingness to pay values obtained in these studies may be overestimates due to the endowment effect. This states that desirable goods are more valuable when they are part of one’s endowment, i.e. individuals put more value on the loss of something they own or have experienced than its acquirement when they have not experienced it [81]. In this situation, health workers may more easily give up hypothetical additional compensation rather than a decrease in their actual salaries. Compensating wage differentials may be more accurate when a level is included in the monetary attribute to represent a decrease in current income, as seen in four studies for at least some participants [5, 47, 70, 82].

More recent studies tended to extend the probability analysis by simulating different policy scenarios, particularly predicting the uptake of jobs in rural areas under different incentive packages. Lagarde *et al.* [54] went further by examining the uptake of rural jobs by Thai doctors under different incentive policies for i) the original population; ii) three hypothetical populations with differing proportions of doctors with rural/urban backgrounds; iii) undergraduate training in Bangkok as opposed to outside the capital. Sivey *et al.* [51] investigated specialty choice for junior doctors in Australia with an unlabelled design consisting of attributes describing various job aspects, but then used data from the accompanying survey sent to all Australian doctors to set typical levels for the same attributes for specialist doctors versus general practitioner (e.g. regular continuity of care for general practitioners). The researchers went on to predict the uptake of general practitioner training under different changes to three policy-amenable attributes: procedural work, academic opportunities, and salary. This study is also the first, to our knowledge, to use revealed preference data from the survey on the proportion of junior doctors actually choosing general practice to calibrate their model, so that the predicted choice probabilities matched the actual choices before starting the policy simulations. This comparison with revealed preference data is to be welcomed [30], although it is rare for DCE practitioners (particularly in LMIC) have access to such comprehensive data.

Five studies combined predictions from a probability analysis with cost data in order to assess the cost impact of favoured policy options [46, 49, 55, 65, 82]. Chomitz *et al.* compared a small number of policy options to improve the maldistribution of doctors in Indonesia with little detail on costings, and reported that bonuses for working in remote or very remote posts would be cheaper to provide than specialist training. In a more detailed analysis, Vujicic *et al.* [82] found that rural allowances would be more cost-effective for attracting nurses to rural posts in Liberia than providing housing or improving equipment. Rao *et al.* [55] showed that reserving postgraduate training places was the most cost-effective policy to encourage both doctors and nurses to take up rural jobs in India, with a higher predicted uptake at a lower cost than salary increases. Lagarde *et al.* [65] combined predicted probabilities from two DCEs, one simulating the current labour market in South Africa and the South African component of the multi-country analysis of policy tools to attract nurses to rural areas [4]. These were used in a Markov model to simulate the distribution of nurses in the labour market over time under different policy scenarios using rural nurse-years as the effectiveness measure. The results showed that salary increases are dominated by non-wage interventions, and “upstream” measures (i.e. recruiting individuals more likely to choose rural posts willingly, such as those with rural upbringings) are more cost-effective than “downstream” interventions, with the most cost-effective policy being the recruitment of students with rural backgrounds.

### Assessment of included studies

**Figure 4:**
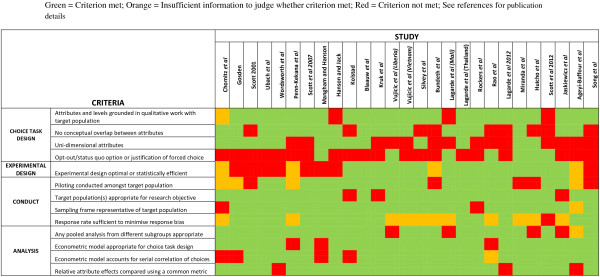
**Validity assessment of included studies.**

Figure 4 presents the validity assessment for all included studies. Overall, whilst the conduct and analysis of studies were more robust than expected, there were significant weaknesses in choice task design. For example, attributes should have no conceptual overlap, i.e. they should be conceptually distinct and vary independently of each other, otherwise their effects are likely to be correlated [5]. For example, Mangham and Hanson [68] excluded the attribute “promotion prospects” that was identified as important in preparatory work because promotion was closely associated with another included attribute “opportunity to upgrade qualifications.” Attributes should also be uni-dimensional, i.e. encompass only one aspect of a characteristic in order to obtain maximum information from the choices made and increase interpretability. Rao *et al.* [55], for instance, included an “Area” attribute that comprised the location’s accessibility, educational facilities for children and the provision of quality housing: from which it would be difficult to unpack the significance of any preferences for this attribute. We identified conceptual overlap in a third of studies and only half of studies had uni-dimensional attributes. This prevalence may be due to the difficulty in reducing complex labour market decisions into a handful of attributes, in comparison to arguably more discrete health products or patient services. However, it should be noted that preparatory qualitative work and piloting receive far less attention in the DCE literature compared to experimental design and analysis, despite their importance in ensuring that choices are salient to the target population and therefore equal contribution to the robustness of results [29, 64].

As discussed above, target populations for HRH studies are often based on logistical factors rather than appropriateness for the research objective. Another important consideration before extrapolating preferences of participants to the general population is the representativeness of the target population. It was anticipated that this would be a particular issue in HRH DCEs, with remote health facilities or rural training schools excluded in preference for more accessible locations. However, the vast majority of sampling frames were found to be representative of target populations. Indeed, national censuses of health workers were quite frequently employed, which likely reflects the overall paucity of health workers in LMIC.

Assessing the validity of experimental design and analytic approach acutely highlights the “moving target” of best practice in DCEs described by Louviere and Lancsar [31]. Studies that employed the best practice at that time are now judged against subsequent advances in the field. For example, a constant comparator was common in earlier studies, although now recognised not to respect level balance and associated with identification problems [31]. Earlier studies also tend not to account for the panel nature of DCE data with serial correlation of choices between the same participants, which can now be adjusted for through an appropriate model or random effects specification. Even recent studies assessed here to have few threats to validity may be judged more critically in a few years, due to the rapid evolution of the field.

### Comparison of results

Out of the 13 studies assessed as meeting more than half the validity criteria, eight had the common objective of determining factors important in the attraction of health workers to rural areas in LMIC and appropriate relative attribute impact analysis available. We used the probability analysis for uptake of a rural post where available (six studies) and willingness-to-pay estimates (two studies) in order to compare preferences for different attributes and their levels (see Additional file 3).

This summary broadly indicates the importance of rural allowances/bonuses and opportunities for further training for the uptake of rural posts, and the unpopularity of time commitments or “bonding”, although it is difficult to conclude further as the range of other included attributes varies widely across studies. Despite using relative analytic measures rather than direct coefficients, such summaries should be treated with caution due to the likely variation in coding practices between studies. Moreover, comparing results from labelled designs to those from generic designs can be problematic as participants may take into account additional, unmeasured factors when comparing labelled alternatives.

## Discussion

There has been a dramatic increase in the number of studies using DCEs to investigate health workforce policy. Twenty-seven studies were identified in this review, with more studies published in the last four years than during 1998–2009. This is the first systematic review of DCEs applied to health workforce policy to our knowledge. Whilst earlier studies may have lagged behind best practice in the field, many of the more recent studies apply state of the art features of design and analysis to address particular issues of health workforces.

Overall, there needs to be more recognition of the heterogeneous nature of health worker experiences, leading to more careful definition of target populations. First, a significant number of studies extrapolated results from students to draw conclusions about the job preferences of qualified health workers. In one study, this even included first year students due to difficulty in recruiting later years [59]. Second, certain study samples included qualified workers with large disparities in professional experience. For example, in one study, the experience of health workers surveyed ranged from 0.42 to 32 years [53]. Previous qualitative research has shown that job preferences of new healthcare graduates are very different from those of even mid- or late-career professionals [83, 84]. Third, several studies pooled the results from different cadres of health workers despite evidence of significant differences in preferences or income (which would affect willingness to pay estimates) [59, 69, 82]. Researchers need to be aware that increasing disparity in professional and life experiences will lead to more heterogeneous job preferences, requiring more sophisticated econometric modelling and more careful interpretation to draw valid conclusions. Such variation may in fact mask any true preferences, negating the value of the research. The expediency of combining groups of health workers to obtain an adequate or convenient sample size is outweighed by the benefits of more robust conclusions for a narrower and well defined study population.

Whilst nearly all studies investigated the relative impact of attributes through willingness-to-pay and/or probability analyses, only five studies went on to combine impact measures with cost data to assess cost-effectiveness of policy options to varying degrees. Just one study to date has used Markov modelling to estimate the cost effectiveness of policies over the long run [65] *.* The paucity of cost effectiveness analysis likely reflects the difficulty in obtaining accurate cost data (direct and indirect) for salaries and other incentives such as training, in addition to the lack of information on career paths to populate a long-term Markov model [65]. This is particularly relevant in LMIC where weak human resource information systems are often a trigger for the use of DCEs over longitudinal studies in the first place. However, cost-effectiveness analysis provides crucial information for policymakers wishing to capitalise on the preferences revealed by DCEs. Indeed, some authors have argued for more use of the willingness to pay values from DCEs in cost-benefit analysis in order to provide fuller evaluation of policy options to decision makers (although concerns have been raised about the use of a price proxy) [30, 85].

All studies included here failed at least some criteria on our validity assessment. This underscores the technical requirements of DCEs for all four stages, but particularly for choice task design. Given that the DCEs reviewed here have been carried out mainly by experienced researchers and that the field is still under great flux, the move to disseminate the use of DCEs more widely amongst non-specialist practitioners may be risky [28].

The strengths of this review include its comprehensive search for studies, both published and unpublished. Virtually all known researchers in this field were contacted in order to identify studies in the grey literature, with seven such studies included in the review. This is also, to our knowledge, the first time that a comparison has been made of results from DCEs in HRH. There may, of course, be other relevant studies not identified through our search strategy. This was also the first attempt to assess the validity of DCEs in order to exclude those with significant potential of bias from the comparison of results. There may be debate over our selection of criteria, although we feel these represent the most important threats to validity over the four stages of DCEs. We welcome further efforts to refine these criteria.

### Implications for research

No study has yet returned to examine how job preferences change over time in the same population. This would provide welcome insights, as would DCEs on a wider range of health workers. Further training after qualification is clearly important to health workers, with over half of designs including such an attribute in some form. Yet no study has yet compared different forms of further training, for example short-term study leave for courses versus specialist training for doctors. Given the necessity of training for career progression for most health workers, it is likely that health workers place different values on various types of training and this could be explored in future research. Lastly, our attempt to compare results of similar studies was limited, despite using more comparable preferences from predicted probabilities and willingness-to-pay estimates. Methodological research on the generalisability and synthesis of results is urgently needed to allow policymakers to make better use of the growing body ofevidence [30].

### Implications for policy

The correlation between health workers’ stated preferences in DCE studies and revealed preferences of longitudinal studies is still uncertain, although one study here made novel use of accompanying survey data to enhance the realism of policy simulations [51]. In other fields, a number of studies show a good correspondence between predictions derived from stated preference models and actual market behaviour [9, 16, 86]. In HRH, this would translate to acceptance of jobs with valued incentive packages or after implementation of preferred policy changes. It is unclear, however, what a discrepancy between stated and revealed preferences would indicate in the case of HRH policy. Willingness to accept a hypothetical post does not always translate into actual acceptance due to many other aspects of policy implementation, imperfect labour market information and life circumstances that can influence a later career decision. What DCEs do provide is constructive information on health worker preferences for exploratory analysis of policy options, thus allowing limited resources to be deployed based on better evidence. Investment into information systems to keep track of health workers and their career choices should not be neglected, however, so that data can be gathered on the impact of implemented policies.

## Conclusions

Discrete choice experiments have become a popular study design to investigate health worker preferences, with several advantages in this field. We identified specific issues relating to this application of which practitioners should be aware to ensure robust results. In particular, there is a need for more defined target populations and increased synthesis with cost data. Research on a wider range of health workers and the generalisability of results would be welcome.

## Electronic supplementary material

Additional file 1: **Criteria to assess validity of included studies.** These are the criteria, with justification, used to assess the validity of studies included in the review. (PDF 181 KB)

Additional file 2: **General characteristics of included studies.** This table summarises key characteristics of the studies included in the review. (PDF 458 KB)

Additional file 3: **Comparison of results for a subset of similar studies.** This is a comparison of the results from relative attribute impact analyses in a subset of studies with low risk of bias and the common objective of investigating health workers’ preferences for jobs in rural areas in low- and middle-income countries. (PDF 385 KB)

## Abbreviations

DCE: Discrete choice experiment
HIC: High-income countries
HRH: Human resources for health
LMIC: Low- and middle-income countries
