# Supplementary material for: The use of discrete choice experiments to inform health workforce policy: a systematic review
Source: BMC Health Serv Res. 2014 Sep 1;14:367. doi: 10.1186/1472-6963-14-367 (PMC4161911; doi:10.1186/1472-6963-14-367)
Supplement: Supplementary file 1 — Additional file 1: Criteria to assess validity of included studies. These are the criteria, with justification, used to assess the validity of studies included in the review. (PDF 181 KB) [file 12913_2014_3466_MOESM1_ESM.pdf]

Additional file 1: Criteria used for assessment of validity of included studies

SECTION	CRITERIA	JUSTIFICATION
**CHOICE TASK DESIGN**	Choice of attributes and levels grounded in qualitative work with target population	Attributes and levels should be salient to the target population to ensure comprehension and engagement with the choice task
	No conceptual overlap between attributes	Attributes should be conceptually distinct and vary independently of each other, otherwise effects will not be independent
	Uni-dimensional attributes	Attributes that encompass several aspects of an attribute introduce variability into the choice process as participants may focus on different aspects and the resulting preferences can only be interpreted as being for all dimensions
	Inclusion of an opt-out or status quo option or justification of forced choice	Choices that force participants to accept an unappealing job are likely to lead to overestimation of preferences
**EXPERIMENTAL DESIGN**	Experimental design optimal or statistically efficient	Designs that are non-optimal or efficient will lead to less accurate preferences
**CONDUCT**	Piloting conducted amongst target population	Validity of choice task design and questionnaire features should be tested with participants from target population and subgroups
	Target population(s) appropriate for research objective	Preferences of target population should be sufficient to answer research objective
	Sampling frame representative of target population	Sampling frames that exclude part of the target population may lead to bias in preferences
	Response rate sufficient to minimise response bias	A low response rate may indicate selection bias amongst participants, whose preferences may not be representative of the target population
**ANALYSIS**	Any pooled analysis from different subgroups appropriate	Pooled analyses from very heterogeneous subgroups may mask marked differences in preferences
	Econometric model appropriate for choice task design	Model should be appropriate for the choice task and number of alternatives presented to participants
	Econometric model accounts for serial correlation of choices	As multiple observations are obtained from each participant, the econometric model should take account of panel nature of data to avoid overestimation of the differences between preferences
	Relative attribute effects compared using a common metric	Preferences for different attributes cannot be compared directly using parameter estimates due to confounding with the underlying utility scales
