# Supplementary material for: The use of discrete choice experiments to inform health workforce policy: a systematic review
Source: BMC Health Serv Res. 2014 Sep 1;14:367. doi: 10.1186/1472-6963-14-367 (PMC4161911; doi:10.1186/1472-6963-14-367)
Supplement: Supplementary file 2 — Additional file 2: General characteristics of included studies. This table summarises key characteristics of the studies included in the review. (PDF 458 KB) [file 12913_2014_3466_MOESM2_ESM.pdf]

Additional file 2: Characteristics of included studies

LEGEND: N/A = Information not available from study publication; GPs = General Practitioners; [a]All information available in report(s) included; [b]848 questionnaires were returned, however 65 were uncompleted therefore response rate taken as 783; [c]Breakdown by subgroup not available; [d]5 records lost, leaving total functional sample size of 302; [e]Medical students excluded from peer-reviewed publication; [f]Total sampling frame of junior doctors not available; [g]Numbers given are for target sample: however, it is unclear whether response rate was 100%; [h]Conference abstract only, not full length paper [i]Also contains data on Thai nurses published in Blauuw *et al*, 2010; [j]Nurses and midwives and nursing/midwifery students analysed as one group in report (33), but only nurses and nursing students included in peer-reviewed publication and analysed as separate groups (34).

Authors & earliest publication date	Peer-reviewed publication	Study Setting	Health worker cadre and type	Sample size (total)	Survey mode[a]	Response rate	Study objective
Chomitz *et al* 1998 (1)	No	Indonesia	Medical students (final year)	585	Self-administered questionnaire supervised by researchers	N/A	To understand doctors' preferences regarding various possible incentives, in particular to attract them to rural or remote places
Gosden 2000 (2)	Yes	England	Doctors (GPs)	172	Self-administered postal questionnaire	58.7% (172/293)	To investigate GP preferences for practice and job characteristics, in order to understand what factors might improve GP recruitment in under-served areas
Scott 2001 (3)	Yes	UK	Doctors (GPs)	783	Self-administered postal questionnaire	65% [b] 783/1206	To investigate GPs' preferences for financial and non-financial incentives
Ubach *et al* 2003 (4)	Yes	Scotland	Doctors (hospital consultants)	1793	Self-administered postal questionnaire	61.3% (1793/2923)	To examine hospital consultants' preferences for various job characteristics to inform workforce policy
Wordsworth *et al* 2004 (5)	Yes	Scotland	Doctors (GPs)	1292	Self-administered postal questionnaire	50.0% (1292/ 2574)	To identify the relative value given by sessional GPs to various job characteristics, in order to inform issues on recruitment and retention of GPs
Penn-Kekana *et al* 2005 (6)	No	South Africa	Nurses (maternity)	147	Self-administered paper-based questionnaire	N/A	To explore the relative importance of various job characteristics on nurses' decisions about where to work
Scott *et al* 2007 (7)	Yes	Scotland	Pharmacists (community)	914	Self-administered postal questionnaire	56.4% (914/1621)	To examine the preferences of community pharmacists for existing and potential new roles in

							primary care
Mangham and Hanson 2008 (8)	Yes	Malawi	Nurses	107	N/A	97.3% (107/110)	To determine the range and relative importance of various factors that affect nurses' job choices in the public sector
Hanson and Jack 2008 (9, 10)	Yes	Ethiopia	Doctors<br><br>Nurses	283<br><br>642<br>**(925)**	Interviewer-administered paper-based questionnaire	70.7% (219/283)<br>N/A for nurses	To estimate the effects of possible policy interventions to improve the supply of doctors and nurses in rural areas
Kolstad 2008 (11, 12)	Yes	Tanzania	Trainee clinical officers (final year)	307	Self-administered paper-based questionnaire supervised by researchers	96% (307/320)	To estimate clinical officers' job preferences in order to understand how rural jobs can be made more attractive
Blaauw *et al* 2010 (13)	Yes	Kenya<br>South Africa<br>Thailand	Nursing students (final year)	345<br>377<br>342<br>**(1064)**	Self-administered paper-based questionnaire supervised by researchers	74.5% (1064/1429)	To determine the relative effectiveness of financial and non-financial strategies to increase nurse recruitment to rural areas
Kruk *et al* 2010 (14)	Yes	Ghana	Medical students (fourth year)	302	Computer-assisted, self-administered questionnaire, supervised by researchers	99.0% (307/310)[d]	To investigate the job attributes that influence medical students' preferences for rural job postings
Vujicic *et al* 2010 (15)	No	Liberia	Nurses (various including nurse anaesthetist and midwives)	197[c]	Interviewer-administered, paper-based questionnaire	N/A	To provide evidence for policy options to retain nurses in rural posts
Vujicic *et al* 2010 (16, 17)	Yes[e]	Vietnam	Doctors<br>Medical students (final year)	292<br>105<br>**(397)**	Interviewer-administered, paper-based questionnaire	N/A	To investigate the preferences of doctors and medical students for rural posts in order to support the development of rural retention policies
Sivey *et al* 2010 (18, 19)	Yes	Australia	Doctors (junior i.e. not entered specialist training)	532	Self-administered postal paper-based or online questionnaire	N/A[f]	To examine the preferences of junior doctors who have not yet entered a specialist training program for different attributes of specialties

Bundeth *et al* 2011 (20, 21)	Yes	Cambodia	Medical students<br>Nursing students<br>Midwifery students<br>(all final year)	82<br>170<br>184<br>**(436)**	Self-administered paper-based questionnaire supervised by researchers	N/A[g]	To explore the financial and non-finanacial incentives required to post new clinical graduates to remote and rural areas
Lagarde *et al* 2011 (22)	No	Mali	Doctors<br>Medical students (final year)<br>Midwives<br>Midwifery students (final year)	116<br>114<br>74<br>102<br>**(396)**	Self-administered paper-based questionnaire supervised by researchers	N/A	To investigate the effect of various policy incentives to attract doctors and midwives into rural and periurban areas
Lagarde *et al* 2011 (23)	Yes	Thailand	Doctors<br>(< 3 years graduated in rural service)	198	Researchers explained questionnaire in person, then participants asked to self-administer questionnaire and return by post	63.3% (198/313)	To measure the preferences of junior doctors for incentives associated with rural/urban jobs
Rockers *et al* 2011 (24, 25)	Yes	Uganda	Medical students<br>Nursing students<br>Pharmacy students<br>Laboratory technician students<br>(all final year)	246<br>132<br>50<br>57<br>**(485)**	Computer-assisted, self-administered questionnaire supervised by researchers	95.0% (246/259)<br>85.2% (132/155)<br>98.0% (50/51)<br>85.1% (57/67)	To investigate the preferences of trainee health workers for job attributes to aid recruitment and retention strategies in rural areas
Rao *et al* 2012 (26, 27)	Yes[h]	India (Andhra Pradesh and Uttarakhandst ates)	Doctors<br>Medical students (final year)<br>Nurses<br>Nursing students (final year)	222<br>163<br>238<br>145<br>**(768)**	Self-administered paper-based questionnaire supervised by researchers	N/A	To examine the effect of monetary and non-incentives on job choices in order to improve recruitment in rural areas
Lagarde *et al* 2012 (28)	Yes[i]	South Africa	Nursing students (final year)	377	N/A	65.2% (377/578)	To investigate the preferences of new nursing graduates for job opportunities in the public sector (rural and urban), the private sector or overseas.

Miranda *et al* 2012 (29)	Yes	Peru	Doctors<br>(on short-term contracts)	102	Interviewer-administered questionnaire in person	N/A	To identify job attributes that would attract doctors to posts in rural areas
Huicho *et al* 2012<br>(30)	Yes	Peru	Nurses<br>Midwives<br>(both on short-term contracts)	205[c]	Interviewer-administered questionnaire in person	N/A	To identify job attributes that would attract nurses and/or midwives to posts in rural areas
Scott et al 2012 (31, 32)	No	Australia	Doctors<br>(GPs)	3727	Self-administered postal paper or online questionnaire	16.8%<br>(3727/22137)	To investigate preferences of GPs for rural locations
Jaskiewicz *et al* 2012<br>(33, 34)[j]	No	Lao People's Democratic Republic	**Graduates:**<br>Doctors<br>Medical assistants<br>Nurses<br><br>Midwives<br><br>**Students:**<br>Medical<br>(5 & 6 year)<br>Medical asssistant<br>(1 & 2 year)<br>Nursing<br>(All years)<br>Midwifery<br>(All years)	 105<br>90<br>249<br><br>40<br><br><br>329<br><br>280<br><br>256<br><br>105<br>**(1454)**	Self-administered paper-based questionnaire, supervised by researchers	 N/A<br>N/A<br>65.0%<br>(249/383)<br>N/A<br><br><br>N/A<br><br>N/A<br><br>62.7%<br>(256/408)<br><br>N/A	To investigate health worker preferences for potential attraction and retention strategies for postings in rural areas
Ageyi-Baffour *et al* 2013<br>(35)	Yes	Ghana	Midwifery students<br>(final year)	238	Computer-assisted, self-administered questionnaire	79.8<br>(238/298)	To understand what would motivate midwifery students to work in rural areas after graduation in order to develop an incentive package
Song *et al* 2013<br>(36)	Yes	China	Doctors<br>Nurses	282<br>235<br>**(517)**	Self-administered supervised by researchers	100%<br>(517/517)	To investigate the job preferences of doctors and nurses working in primary care

References for Additional File 2

1. Chomitz K, Setiadi G, Azwar A, Ismail N, Widiyarti. What Do Doctors Want? Developing Incentives for Doctors to Serve in Indonesia's Rural and Remote Areas. Policy Research Working Paper Series No. 1888. Washington, D.C.: World Bank; 1998.
2. Gosden T, Bowler I, Sutton M. How do general practitioners choose their practice? Preferences for practice and job characteristics. Journal of Health Services Research and Policy. 2000;5:208-13.
3. Scott A. Eliciting GPs' preferences for pecuniary and nonpecuniary job characteristics. Journal of Health Economics. 2001;20:329-47.
4. Ubach C, Scott A, French F, Awramenko M, Needham G. What do hospital consultants value about their jobs? A discrete choice experiment. British Medical Journal. 2003;326:1432.
5. Wordsworth S, Skatun D, Scott A, French F. Preferences for general practice jobs: a survey of principals and sessional GPs. Journal of General Practice. 2004;54:740-6.
6. Penn-Kekana L, Blaauw D, San Tint K, Monareng D, Chege J. Nursing staff dynamics and implications for maternal health provision in public health facilities in the context of HIV/AIDS. Johannesburg: Centre for Health Policy, University of Witwatersrand; 2005.
7. Scott A, Bond C, Inch J, Grant A. Preferences of community pharmacists for extended roles in primary care: a survey and discrete choice experiment. Pharmacoeconomics. 2007;25:783-92.
8. Mangham L, Hanson K. Employment preferences of public sector nurses in Malawi: results from a discrete choice experiment. Tropical Medicine and International Health. 2008;13(12):1-9.
9. Hanson K, Jack W. Health worker preferences for job attributes in Ethiopia: Results from a discrete choice experiment. Washington, D.C.: World Bank; 2008.
10. Hanson K, Jack W. Incentives Could Induce Ethiopian Doctors And Nurses To Work In Rural Settings. Health Affairs. 2010;29(8):1452-60.
11. Kolstad JR. How to make rural jobs more attractive to health workers. Findings from a discrete choice experiment in Tanzania. Working Papers in Economics No 15/08, Bergen: Department of Economics, University of Bergen; 2008.
12. Kolstad J. How to make rural jobs more attractive to health workers. Findings from a discrete choice experiment in Tanzania. Health Economics. 2010;20(2):196-211.
13. Blaauw D, Erasmus E, Pagaiya N, Tangcharoensathein V, Mullei K, Mudhune S, et al. Policy interventions that attract nurses to rural areas: a multicountry discrete choice experiment. Bulletin of the World Health Organization. 2010;88:350-6.
14. Kruk M, Johnson J, Gyakobo M, Agyie-Baffour P, Asabir K, Kotha S, et al. Rural Practice Preferences among Medical Students in Ghana: a Discrete Choice Experiment. Bulletin of the World Health Organization. 2010;88:333-41.
15. Vujicic M, Alfano M, Ryan M, Sanford Wesse C, Brown-Annan J. Policy options to retain nurses in rural Liberia: Evidence from a discrete choice experiment. Washington, D.C.: World Bank; 2010.
16. Vujicic M, Alfano M, Shengeliac B, Witter S. Attracting Doctors and Medical Students to Rural Vietnam: Insights from a Discrete Choice Experiment. Washington, D.C.: World Bank; 2010.
17. Vujicic M, Shengelia B, Alfano M, Bui Thu H. Physician shortages in rural Vietnam: Using a labor market approach to inform policy. Social Science & Medicine. 2011;73:970-7.
18. Sivey P, Scott A, Witt J, Humphreys J, Joyce C. Why Junior Doctors Don't Want to Become General Practitioners: A Discrete Choice Experiment from the MABEL Longitudinal Study of Doctors. Melbourne Institute Working Paper No 17/10. Melbourne, University of Melbourne: 2010.
19. Sivey P, Scott A, Witt J, Joyce C, Humphreys J. Junior doctors' preferences for specialty choice. Journal of Health Economics. 2012;31:813-23.
20. Bundeth S, Neath N, Nognlak P, Sethea S. Incentives and the retention of health workers in rural and disadvantaged areas of Cambodia. CDRI Annual Development Review 2010-11. Phnom Penh: Cambodia Development Research Institute; 2011.

21. Bundeth S, Neath N, Nonglak P, Sethea S. Understanding rural health service in Cambodia: results of a discrete choice experiment. Improving Health Sector Performance: Institutions, Motivations and Incentives. Singapore: Institute of Southeast Asian Studies; 2011.
22. Lagarde M, Traore O, Juquois M, Lemiere C. Quelles politiques incitatives pour attirer le personnel de sante malien hors de Bamako? Resultats du questionnaire emploi DCE Mali. Washington, D.C.: World Bank; 2011.
23. Lagarde M, Pagaiya N, Tangchareonsathien V, Blaauw D. One size does not fit all: investigating doctors' stated preference heterogeneity for job incentives to inform policy in Thailand. Health Economics. 2013;22(12):1452-69.
24. Rockers P, Jaskiewicz W, Wurts L, Mgomella G. Determining Priority Retention Packages to Attract and Retain Health Workers in Rural and Remote Areas in Uganda. Washington, D.C.: Capacity*Plus*; 2011.
25. Rockers P, Jaskiewicz W, Wurts L, Kruk ME, Mgomella G, Ntalazi F, et al. Preferences for working in rural clinics among trainee health professionals in Uganda: a discrete choice experiment. BMC Health Services Research. 2012;12: 212.
26. Rao KD, Shroff Z, Ramani S, Khandpur N, Murthy S, Hazarika I, et al. How to Attract Health Workers to Rural Areas? Findings from a Discrete Choice Experiment from India. Washington, D.C.: World Bank; 2012.
27. Rao KD. How to attract health workers to rural areas? Findings from a Discrete Choice Experiment in India. BMC Proceedings. 2012;6 (suppl 5):O1.
28. Lagarde M, Blaauw D, Cairns J. Cost-effectiveness analysis of human resources policy interventions to address the shortage of nurses in rural South Africa. Social Science & Medicine. 2012;75(5):801-806.
29. Miranda JJ, Diez-Canseco F, Lema C, Lescano AG, Lagarde M, Blaauw D, et al. Stated Preferences of Doctors for Choosing a Job in Rural Areas of Peru: A Discrete Choice Experiment. PLoS ONE. 2012;7(12):e50567.
30. Huicho L, Miranda JJ, Diez-Canseco F, Lema C, Lescano AG, Lagarde M, et al. Preferences of Nurses and Midwives for Taking Up a Rural Job in Peru: A Discrete Choice Experiment. PLoS ONE. 2012;7(12):e50315.
31. Scott A, Witt J, Humphreys J, Joyce C, Kalb G, Jeon S-H, et al. Getting Doctors into the Bush: General Practitioners' Preferences for Rural Location. Melbourne Institute Working Paper Series. Working Paper No. 13/12. Melboune: University of Melbourne; 2012.
32. Scott A, Witt J, Humphreys J, Joyce C, Kalb G, Jeon S-H, et al. Getting doctors into the bush: General Practitioners' preferences for rural location. Social Science & Medicine. 2013;96:33-44.
33. Jaskiewicz W, Phathammavong O, Vangkonevilay P, Paphassarang C, Phachanh IT, Wurts L. Toward Development of a Rural Retention Strategy in Lao People's Democratic Republic: Understanding Health Worker Preferences. Washington, D.C.: Capacity*Plus*; 2012.
34. Rockers P, Jaskiewicz W, Kruk M, Phathammavong O, Vanghonevilay P, Paphassarang C, et al. Differences in preferences for rural job postings between nursing students and practicing nurses: evidence from a discrete choice experiment in Lao People's Democratic Republic. Human Resources for Health. 2013;11:22.
35. Ageyi-Baffour P, Rominski S, Nakua E, Gyakobo M, Lori JR. Factors that influence midwifery students in Ghana when deciding where to practice: a discrete choice experiment. BMC Medical Education. 2013;13:64.
36. Song K, Scott A, Sivey P, Meng Q. Improving Chinese primary care providers' recruitment and retention: a discrete choice experiment. Health Policy and Planning. 2013:doi: 10.1093/heapol/czt098.
