# Supplementary material for: The use of discrete choice experiments to inform health workforce policy: a systematic review
Source: BMC Health Serv Res. 2014 Sep 1;14:367. doi: 10.1186/1472-6963-14-367 (PMC4161911; doi:10.1186/1472-6963-14-367)
Supplement: Supplementary file 3 — Additional file 3: Comparison of results for a subset of similar studies. This is a comparison of the results from relative attribute impact analyses in a subset of studies with low risk of bias and the common objective of investigating health workers’ preferences for jobs in rural areas in low- and middle-income countries. (PDF 385 KB) [file 12913_2014_3466_MOESM3_ESM.pdf]

**Additional file 3: Comparison of results for a subset of studies with common objective of investigating health workers' preference for jobs in rural areas in low- and middle-income countries)**

VND = Vietnamese dong; USD = United States dollar; Tied rankings indicated by "="; See References in main text for details of publications; *Rockers *et al* 2013 presents willingness to pay estimates for single interventions for nurses and nursing students only, whereas Jaskiewicz *et al* presents only packages of interventions

<table>
<tr><th>Authors*</th><th>Analytic approach</th><th colspan="3">Results of relative attribute impact analysis<br>(ranked from most preferred to least preferred)</th></tr>
<tr><td>Hanson and Jack</td><td>Marginal willingness to pay</td><td colspan="2">Doctors:<br>Private practice<br>Superior housing<br>Job in Addis Ababa (national capital)<br>Adequate equipment<br>Two-year time commitment</td><td>Nurses:<br>Job in zonal capital<br>Adequate equipment<br>Superior housing<br>Improved supervision<br>Two-year time commitment</td></tr>
<tr><td>Blaauw et al</td><td>Predicted probabilities of uptake of rural post under different incentives</td><td>Kenya:<br>30% rural allowance<br>20% rural allowance<br>Preferential training opportunities<br>More rapid promotion<br>10% rural allowance<br>Better rural housing<br>Relational management culture<br>Benefit package</td><td>South Africa:<br>30% rural allowance<br>Preferential training opportunities<br>20% rural allowance<br>10% rural allowance<br>Benefit package<br>More rapid promotion<br>Better rural housing<br>Relational management culture</td><td>Thailand:<br>Benefit package<br>Relational management culture<br>More rapid promotion<br>30% rural allowance<br>Better rural housing<br>20% rural allowance<br>10% rural allowance<br>Preferential training opportunities</td></tr>
<tr><td>Kruk et al</td><td>Predicted probabilities of uptake of rural post under different incentives</td><td colspan="3">Improved infrastructure<br>100% salary increase<br>Supportive management<br>Study leave after 2 years<br>Utility car provided<br>Allowance for children's education<br>Superior housing</td></tr>
</table>

<table>
<tr><td></td><td></td><td colspan="4">50% salary increase<br>30% salary increase<br>No housing (compared to basic housing)</td></tr>
<tr><td>Vujicic et al<br>(Vietnam)</td><td>Predicted probabilities of uptake of rural post under different incentives</td><td colspan="4">VND 3 million financial bonus<br>VND 2 million financial bonus = Provision of long-term education = Improvement of equipment<br>Provision of short-term training<br>VND 1 million financial bonus<br>Provision of housing<br>VND 500,000 financial bonus</td></tr>
<tr><td>Vujicic et al<br>(Liberia)</td><td>Predicted probabilities of uptake of rural post under different incentives</td><td colspan="4">Increase pay by USD150<br>Increase pay by USD100<br>Provide housing<br>Provide transportation<br>Increase pay by USD50<br>Improve equipment<br>Increase pay by USD25</td></tr>
<tr><td>Lagarde et al<br>(Thailand)</td><td>Predicted probabilities of uptake of rural post under different incentives</td><td colspan="4">Workplace close to home province<br>Place in quota for specialty training<br>45% rural incentive<br>Consultant supervision<br>On-call 7 nights per month (compared to 14 nights)<br>1 year before promotion (compared to two)<br>30% rural incentive</td></tr>
<tr><td>Rockers et al<br>2012</td><td>Marginal willingness to pay</td><td>Medical students:<br>Tuition support for training<br>Advanced facility quality<br>2 year commitment<br>Housing allowance<br>Housing provided<br>Supportive management</td><td>Nursing students:<br>Advanced facility quality<br>Supportive management<br>Housing allowance = Housing provided<br>Full staff levels<br>25% understaffed<br>2 year commitment</td><td>Pharmacy students:<br>Ownership of private pharmacy<br>Housing provided<br>Housing allowance<br>Advanced facility quality<br>Supportive management<br>2 year commitment</td><td>Laboratory students:<br>Advanced facility quality<br>Tuition support for training<br>Housing provided<br>Housing allowance<br>Supportive management<br>2 year commitment</td></tr>
</table>

| | | |
|---|---|---|
| Miranda *et al* | Predicted probabilities of uptake of rural post under different incentives | 75% rural allowance<br>50% rural allowance<br>Permanent contract after 2 years<br>20 points for specialisation<br>25% rural allowance<br>Permanent contract after 4 years<br>10 points for specialisation |
